# PD184 Health Technology Assessment (HTA) Topics That Respond To National Needs: Considerations Around The Topic Selection Process When Institutionalizing HTA

**DOI:** 10.1017/S0266462324004069

**Published:** 2025-01-07

**Authors:** Elizabeth Peacocke, Lieke Heupink, Aparna Ananthakrishnan, Katrine Frønsdal

## Abstract

**Introduction:**

Most HTA processes follow similar institutional mechanisms, starting with topic selection and prioritization, followed by analysis (appraisal, deliberation, and decision-making) and implementation. The process of conducting an HTA is financially and time intensive. Therefore, to sustain HTA decision-makers, especially in countries with limited capacity, selecting topics for HTA that most respond to national needs is crucial.

**Methods:**

Information on topic identification, selection, and prioritization (TISP) processes was taken from a recent report published by the Norwegian Institute of Public Health (NIPH) on how to support capacity building for HTA in low- and middle-income countries. An unpublished survey of 29 national HTA organizations around the world was also performed by the NIPH asking about their TISP processes. Issues around the institutional and organizational aspects necessary for explicit and transparent TISP processes were identified and discussed through an iterative process.

**Results:**

The comprehensiveness of TISP processes varied according to each country’s needs and the types of decisions supported by HTA. Accordingly, the resources available for allocation within the health system, the number of dedicated personnel available to complete HTAs, and the number stakeholders and institutions involved in the decision-making process may all be relevant considerations for TISP. In countries where HTA was well-established, the process for TISP was usually institutionalized or at least somewhat formalized. In settings where HTA was emerging or relatively new, or where there may not be the necessary supporting institutional mechanisms, there was limited normative guidance on how to implement TISP.

**Conclusions:**

When institutionalizing HTA, we argue for including formal and explicit processes for the topic selection step that include: (i) a clear link to health system feasibility; (ii) process transparency to ensure legitimacy and impact; and (iii) patient and public engagement. Insights and experiences from countries with more formalized HTA systems can provide valuable lessons.

